# Algae and Algal Protein in Human Nutrition: A Narrative Review of Health Outcomes from Clinical Studies

**DOI:** 10.3390/nu18020277

**Published:** 2026-01-15

**Authors:** Zixuan Wang, Marie Scherbinek, Thomas Skurk

**Affiliations:** 1 Core Facility Human Studies, ZIEL—Institute for Food & Health, Technical University of Munich, Gregor-Mendel-Str. 2, 85354 Freising, Germany; 2 Klinikum Rechts der Isar, School of Medicine and Health, Technical University of Munich, Ismaninger Straße 22, 81675 München, Germany

**Keywords:** human nutrition, algal protein, alternative protein source, microalgae, human studies

## Abstract

As global interest in sustainable nutrition grows, algae have emerged as a promising functional food resource. This review analyzes the nutritional value of edible algae, with a particular focus on protein-rich microalgae, and synthesizes current clinical evidence regarding their health benefits. Algae have been demonstrated to provide a broad spectrum of physiologically active nutrients, encompassing a range of vitamins and minerals as well as polyunsaturated fatty acids, antioxidant molecules and various bioactive compounds including dietary fiber. These nutrients have been linked to improved cardiovascular and metabolic health, enhanced immune function, and anti-inflammatory effects. A particular emphasis is placed on algal proteins as a novel alternative to traditional dietary proteins. Genera such as *Spirulina* and *Chlorella* offer high-quality, complete proteins with amino acid profiles and digestibility scores comparable to those of animal and soy proteins, thereby supporting muscle maintenance and overall nutritional status. Recent clinical studies have demonstrated that the ingestion of microalgae can stimulate muscle protein synthesis and improve lipid profiles, blood pressure, and inflammation markers, indicating functional benefits beyond basic nutrition. Algal proteins also contain bioactive peptides with antioxidative properties that may contribute to positive outcomes. This review synthesizes current studies, which demonstrate that algae represent a potent, sustainable protein source capable of enhancing dietary quality and promoting health. The integration of algae-based products into plant-forward diets has the potential to contribute to global nutritional security and long-term public health. However, the available clinical evidence remains heterogeneous and is largely based on small, short-term intervention studies, with substantial variability in algae species, processing methods and dosages. Consequently, while the evidence suggests the possibility of functional effects, the strength of the evidence and its generalizability across populations remains limited.

## 1. Introduction

The global food production system is confronted with significant challenges, primarily due to an ever-growing global population, compounded by climate change and the depletion of natural resources. It is estimated that global food production must increase by 70% by the year 2050 to meet the nutritional needs of the projected future population [1]. Protein, an essential macronutrient, is anticipated to be in short supply in the future. Consequently, there is an increasing interest in identifying novel, sustainable, and nutrient-dense protein sources [2]. Among the various candidates under consideration, algae are particularly promising, offering both considerable health benefits and meaningful environmental advantages. Owing to their robust nutritional profiles and diverse bioactive compounds, algae have gained significant attention in clinical research.

Algae are photosynthetically active aquatic organisms that exhibit significant polyphyletic diversity. These organisms can be broadly categorized into two distinct groups: microalgae and macroalgae. Microalgae, including species such as *Chlorella* and *Spirulina*, are microscopic unicellular organisms. Macroalgae, commonly referred to as seaweeds, are multicellular complex organisms, such as kelps. It is important to note that *Spirulina*, as well as *Aphanizomenon flos-aquae* (AFA), are taxonomically classified as cyanobacteria. However, they are historically and functionally grouped with microalgae due to their similar morphology, photosynthetic activity, and nutritional profile. Both microalgae and seaweeds are nutritionally dense, with substantial protein contents and a complete profile of essential amino acids. Moreover, they also provide a wide array of vitamins and minerals, in addition to long- and short-chain polyunsaturated fatty acids (PUFAs) and complex polysaccharides. Certain species of algae are known to contain protein levels (e.g., *Chlorella pyrenoidosa* 57–60% of dry weight DW) comparable to those of conventional protein sources, such as egg and beef (e.g., egg 53% of DW) [2,3,4].

**Figure 1:**
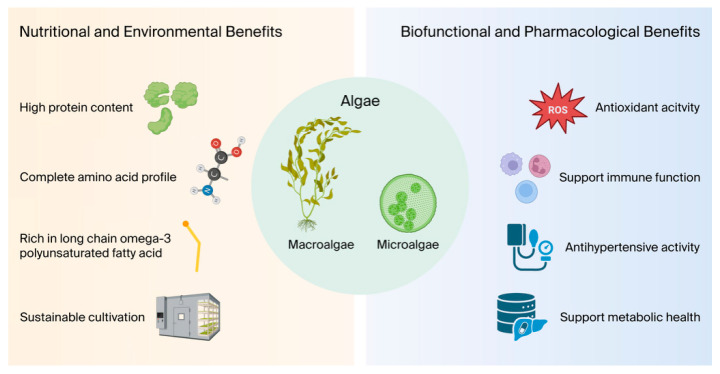
Overview of algae benefits, including nutritional, environmental, biofunctional and pharmacological effects (Created in BioRender. Zixuan W. (2025) https://doi.org/10.5281/zenodo.18243464. Icons used under Biorender’s Academic License).

Beyond their nutritional value, algae are recognized for producing a wide range of bioactive compounds, which are secondary metabolites with diverse physiological effects (Figure 1). These compounds include polysaccharides, carotenoids, polyphenols, and phycobiliproteins. The distinctive chemical composition of algae is considered to have significant clinical potential, thus prompting numerous studies exploring their application in the management of conditions such as cardiovascular diseases, metabolic disorders, and cancer [5].

This literature review aims to provide a comprehensive overview of the current state of clinical trials involving algae. It will examine the nutritional benefits as well as the therapeutic potential of various species of algae and algae-derived products. By critically evaluating the available evidence, this review seeks to highlight future directions and opportunities for the use of algae in both nutritional science and clinical medicine, with a particular focus on their potential as sustainable protein sources with functional health effects.

## 2. Nutritional Values

Algae have a long history of incorporation into indigenous cuisines in Asian countries such as Japan and China. However, due to their pronounced fishy flavor, algae are used sparingly in culinary applications, primarily as a seasoning or for nori wrappings. Their abundant content of fiber, vitamins, and protein supports the potential utilization in future novel foods, thereby offering health benefits while contributing to sustainable food sources. In order to achieve a successful outcome, it is imperative to implement measures that mitigate sensory barriers, such as the unpleasant fishy taste. Among these nutrients, protein is a key component that contributes to the nutritional value of algae due to its abundance and importance in human nutrition. However, the protein content can vary between species. Macroalgae (seaweed) generally have lower protein content compared to microalgae. Among seaweeds, brown seaweed has the lowest protein content, averaging 15.9% of DW. In contrast, red and green seaweed have higher protein content, reaching up to 32.3% and 28.7% of DW, respectively [6]. Microalgae, in particular *Spirulina platensis* and *Chlorella vulgaris*, consistently demonstrate higher protein contents (up to 70% of DW) compared to most macroalgae species. Their amino acid composition and digestibility contribute to their classification as high-quality proteins, as discussed in the following section on the health relevance of algal proteins.

In addition to protein, other bioactive compounds found in algae, such as polysaccharides and carotenoids, have been shown to exert various beneficial effects on human health. A study by García et al. investigated the effects of microalgae *Tetraselmis chuii* supplementation on multiple parameters in healthy young men. The findings indicated substantial increases in anthropometric parameters, including percent muscle mass, as well as humoral parameters, such as insulin-like growth factor, and hematological parameters, including lymphocytes levels. Moreover, a reduction in body fat mass, platelet count, hematocrit, and mean corpuscular hemoglobin (MCH) was observed, suggesting an overall health benefit from the supplementation [7]. In elderly subjects, dietary supplementation with the microalgae *Phaeodactylum tricornutum* has been demonstrated to enhance mobility parameters, such as five-second sit-to-stand test and gait speed. In addition, plasma Interleukin (IL)-6 levels were found to decrease, suggesting an anti-inflammatory effect of the supplement [8]. Polysaccharides such as alginate and carrageenan, which are primarily found in algae, have garnered attention for their immunomodulatory, antiviral, and anticoagulant activities. Oral supplementation of algal sulfated polysaccharides extracted from *Ulva* sp. has been demonstrated to decrease levels of inflammatory makers, including interferon-γ (IFN-γ), IL-1β and tumor necrosis factor (TNF). Additionally, there was also a shift in the gut microbiome and improvements in plasma lipid profiles in specific participants [9].

Furthermore, several studies have validated the bioavailability of iodine from seaweed, potentially influencing thyroid functions in humans [10,11,12,13].

## 3. Protein Quality and Health Effects of Algal Proteins

*Spirulina platensis* and *Chlorella vulgaris* are two species of microalgae that are among the richest natural protein sources. These organisms have been found to contain protein levels that can reach 60–70% of DW, which is significantly higher than the protein content of most plant-based foods (e.g., soybeans range from 36–40% of DW) [14]. In addition, these algal proteins provide all essential amino acids in proportions comparable to the FAO/WHO recommendations for human nutrition [2]. This comprehensive amino acid profile contrasts with many conventional plant proteins (e.g., legumes, grains) that are often limited in one or more essential amino acids. These findings underscore the potential of algae to complement diverse dietary needs.

Notably, the protein quality of *Spirulina* and *Chlorella* is in a range similar to that of high-quality animal and soy proteins (Table 1). They achieve protein digestibility-corrected amino acid scores (PDCAASs) ranging from 0.75 to 1.0, depending on the processing method and strain, thus approaching the benchmarks set by egg and soy [15,16]. These values indicate a high biological value and digestibility, affirming that microalgae can deliver protein efficiently. Although human data regarding the digestible indispensable amino acid scores (DIAASs) for algal foods are not yet available, preliminary findings from animal models suggest that certain microalgae, such as *Spirulina platensis* and *Pavlova* sp. 459, have the potential to achieve a DIAAS > 1 [17]. This underscores their promise as a valuable protein source.

Beyond their amino acid composition, algal proteins are highly digestible and yield a variety of bioactive peptides. These peptides, released during gastrointestinal proteolysis or food processing, have demonstrated a variety of bioactivities in vitro and in animal studies, including antihypertensive, antioxidative, immunomodulatory, and anti-inflammatory effects. For instance, peptides derived from *Spirulina platensis* have demonstrated potent angiotensin-converting enzyme (ACE)-inhibitory activity, suggesting a mechanism for blood pressure reduction [18]. Although direct evidence from human trials on isolated algal peptides remains limited, the intrinsic presence of these functional peptides contributes to the health-promoting potential of algal protein. In accordance with these mechanistic observations, some clinical studies employing whole algae have documented anti-inflammatory outcomes. Notably, a trial in older adults reported that supplementation with eicosapentaenoic acid (EPA)-rich microalgae led to a significant decrease in IL-6 levels, indicating a reduction in systemic inflammation. Furthermore, a recent meta-analysis of randomized trials found that *Spirulina* intake was associated with a significant decrease in C-reactive protein (a key inflammatory marker) compared to control [19]. These findings suggest that regular consumption of algal products may offer anti-inflammatory benefits in humans, potentially mediated by their bioactive protein components and associated micronutrients.

One of the most compelling aspects of algal protein is its potential to support muscle protein synthesis (MPS) and lean body mass, which is crucial for metabolic health and the prevention of age-related muscle loss. Recent clinical evidence indicates that algal proteins can effectively stimulate muscle anabolic processes. In a randomized controlled trial, ingestion of a single acute dose of 25 g protein from *Chlorella* or *Spirulina* resulted in postprandial increases in blood essential amino acids and robust stimulation of myofibrillar protein synthesis over a 4 h postprandial period in both resting and exercised muscle. This degree of stimulation was comparable to that of an equivalent dose of high-quality animal-derived protein [20]. This acute anabolic response suggests that microalgae-derived proteins could serve as a viable plant-based alternative to traditional proteins, such as dairy or soy in supporting muscle remodeling and maintenance [20]. Consistent with these findings, algae supplementation has shown promise in populations with elevated protein requirements. Additionally, preliminary studies in athletic contexts report that the addition of microalgae to the diet may enhance exercise performance and recovery. In one study, endurance athletes receiving microalgae *Tetraselmis chuii* or *Arthrospira platensis* exhibited improved oxygen uptake and hematological alterations. However, the precise nature of these effects, whether they are attributed to algal protein itself or to other algal bioactive components, remains to be elucidated [21,22].

In patients diagnosed with fibromyalgia, a chronic musculoskeletal disorder of unknown etiology, the administration of nutritional supplementation with *Chlorella* helped relieve symptoms in several participants [23].

The influence of algal proteins and peptides on immune and metabolic health extends beyond their role in muscle function. Some amino acids and peptides derived from algae have immunomodulatory properties, which have the potential to enhance host defenses. Human trials have noted that chronic *Chlorella* supplementation can enhance certain immune markers. For example, in adults undergoing intensive training, *Chlorella* intake attenuated the usual exercise-induced drop in salivary secretory immunoglobulin A (IgA, an important antibody in mucosal immunity), and short-term supplementation led to elevated resting IgA levels. These findings suggest that the observed improvement in immune alertness may offer enhanced protection against infections, particularly under physical stress. There is also evidence that algae may improve vaccine responses. Specifically, supplements derived from *Chlorella* have been associated with higher antibody titers following influenza vaccination in healthy adults [24].

In addition, algal consumption has been examined in the context of metabolic and cardiovascular health. While there is insufficient evidence to confirm the direct impact of algal proteins on lowering blood lipids, the incorporation of microalgae into the diet may indirectly benefit cardiovascular health through the improvement of overall diet quality and the provision of bioactive compounds. Several controlled studies conducted on individuals diagnosed with mild hyperlipidemia have indicated that daily *Chlorella* intake results in a modest reduction in total and LDL-cholesterol and triglycerides (TG), alongside increases in beneficial HDL-cholesterol. These effects are thought to result from algae’s fiber, antioxidant, and omega-3 content in addition to protein. Algal peptides might also contribute to cardiovascular benefits via blood pressure reduction: a 12-week trial showed that a peptide-enriched *Chlorella* extract (rich in γ-aminobutyric acid) significantly lowered systolic blood pressure in adults with borderline hypertension [25]. Collectively, these outcomes illustrate the multifaceted health impacts of algae-derived nutrients—from strengthening immune functions to attenuating risk factors for chronic diseases.

## 4. Cardiovascular Health and Lipoprotein Metabolism

Cardiovascular disease (CVD) is one of the leading causes of global morbidity and mortality. The primary risk factors associated with CVD include elevated levels of blood cholesterol, TG levels, and chronic inflammation [26]. Bioactive compounds, such as polyphenols derived from algae have gained particular interest for their potential to moderate these risk factors. While algal proteins themselves have not yet been directly associated with lipid-lowering effects, they may indirectly support cardiovascular health by contributing to overall dietary protein quality in plant-based diets. For instance, algae have been shown to contain high quality, complete proteins, including all essential amino acids. These amino acids have been demonstrated to play a crucial role in immune function and may therefore contribute to the maintenance of long-term cardiovascular health.

Growing evidence suggests a correlation between low intake of long-chain omega-3 polyunsaturated fatty acids (LCn-3 PUFAs), particularly eicosapentaenoic acid (EPA) and docosahexaenoic acid (DHA), and an elevated risk of cardiovascular diseases [27]. These fatty acids have been shown to effectively reduce blood triglyceride levels in both healthy individuals and those with hypertriglyceridemia [28]. Seafood, particularly fatty fish, is regarded as the primary dietary source of LCn-3 PUFAs, including EPA and DHA. The vast majority of EPA and DHA supplements that are currently available on the market are derived from fish. However, the lack of seafood in certain Western and vegan diets frequently results in an inadequate intake of LCn-3 PUFAs, which may elevate the risk of developing CVD [27,29,30]. Algae which contain high levels of LCn-3 PUFAs present a novel solution to this problem. Consequently, numerous clinical trials have investigated the effects of algal supplements on cardiovascular health, with a particular focus on their LCn-3 PUFA content.

Several trials (Table 2) have demonstrated the optimal bioavailability of LCn-3 PUFAs after consuming algae or algae extracts. DHA supplements derived from microalgae, *Schizochytrium* sp. have been shown to increase serum DHA levels in healthy adults following different diets (omnivores, vegetarian, and vegan). The effect was observed across all three dietary groups, with the most pronounced increase seen in vegans [29]. Similarly, the supplementation of *Ulkenia* sp. oil [30], *Phaeodactylum tricornutum* [31] and *Chlorella* [32] has been shown to result in an increase in LCn-3 PUFA levels.

In accordance with these findings, a study conducted by Rao et al. demonstrated that ethanol extracts from the microalgae *Nannochloropsis* enhanced the EPA level and omega-3 index (O3I, the concentration of EPA and DHA in the membrane of erythrocytes relative to total fatty acids) after 12 weeks of supplementation in healthy participants. Furthermore, the algae supplementation resulted in a decrease in cardiometabolic markers, including total cholesterol (TC) and very-low-density lipoprotein cholesterol (VLDL-C), thereby underscoring its beneficial effects on cardiovascular health. However, no substantial changes in triglyceride or low-density lipoprotein cholesterol (LDL-C) levels were detected [27]. In contrast, a trial by Geppert et al. reported an increase in LDL-C levels alongside a reduction in TG after the administration of a DHA-rich and almost EPA-free oil derived from the microalgae *Ulkenia* sp. [33]. Reflecting the variability of results, the administration of a powdered extract from the brown seaweed *F. vesiculosus* did not significantly alter most biomarkers, with the exception of an increase in high-density lipoprotein cholesterol (HDL-C) [26].

In patients diagnosed with hypertriglyceridemia, the administration of microalgae *Schizochytrium* sp. Oil, which is rich in DHA and EPA, led to a significant reduction in TG levels. However, it also led to a notable increase in LDL-C levels, which may be attributed to the downregulation of the LDL-receptor [28]. Conversely, *Chlorella* supplementation, as reported by Ryu et al., has been shown to significantly reduce TG, TC and VLDL-C in mildly hypercholesterolemic individuals [34]. Kim et al. evaluated the lipid-regulating potential of *Chlorella* in the context of excess dietary cholesterol intake. Their findings indicated the Chlorella supplementation exerted a preventive effect by increasing HDL-C levels and mitigating the rise in TC and LDL-C levels [35].

It has been proposed that the ratio of DHA to EPA in nutritional supplements may be a contributing factor to the observed variability in the effects on the level of lipoprotein. For instance, DHA formulations appear to increase LDL-C levels in response to a reduction in VLDL-C levels whereas EPA formulations seem to decrease VLDL-C levels without influencing LDL-C levels [27]. Although the overall beneficial effect of algae supplements in providing LCn-3 PUFAs is evident, conflicting results regarding the impact of algae on lipoprotein levels require further investigation.

Another key risk factor for CVD is hypertension (Table 3). *Chlorella* with γ-aminobutyric acid (GABA) has demonstrated anti-hypertensive effects in adult subjects with high-normal blood pressure and borderline hypertension. A 12-week administration of GABA-rich *Chlorella* significantly reduced systolic blood pressure, while diastolic blood pressure showed a decreasing trend [25]. However, a recent meta-analysis by Pinto-Leite et al. reported contradictory findings. The analysis of 12 studies revealed that *Chlorella* supplementation exhibited no significant effect on blood pressure, lipid profiles, or body composition, when compared to placebo. In contrast, *Spirulina* supplementation (9 studies) produced a minor but statistically significant change in diastolic pressure (−0.42 mmHg) [36]. In addition, macroalgae *Pyropia yezoensis* and *Undaria pinnatifida* have demonstrated blood-pressure lowering effects in young Japanese boys and hypertensive patients, respectively [37,38].

## 5. Weight Management and Metabolic Health

Obesity is associated with a multitude of chronic diseases and comorbidities including diabetes mellitus and musculoskeletal disorders. The global prevalence of obesity is a matter of significant concern, affecting approximately 10% of the adult population. In Westernized countries, obesity is one of the major public health concerns. For example, in the United States, 40% of the adults were classified as obese in 2023 [39]. Contributing factors to overweight and obesity include physiological changes, such as bone mass loss, lack of physical activity, and unhealthy dietary habits. Weight management interventions generally consist of a combination of physical exercise and dietary modifications to address these issues.

In addition to pharmacological approaches, the effect of various healthy foods, including algae, against excessive weight gain have been extensively studied (Table 4). One species of red seaweed, *Gelidium elegans* (*G. elegans*), has been reported to reduce body weight and body mass index (BMI) following 12 weeks of dietary supplementation (1000 mg extract per day) [40]. Notably, reductions in total body fat mass and visceral abdominal fat were observed. In diet-induced obese mice, *G. elegans* has been shown to downregulate adipogenic transcription factors, such as PPARγ, C/EBPα, and SREBP-1 [30,31,32]. The same mechanisms are proposed for humans. These effects may be attributed to the ability of *G. elegans* to suppress adipocyte differentiation [40].

In overweight women, carotenoids extracted from microalgae *Phaeodactylum tricornutum*, such as fucoxanthin, have been shown to provide additional benefits when combined with a diet and exercise program. The findings indicated that the intervention was effective in preserving bone mass, enhancing bone density, and leading to greater improvements on walking steps [41]. Moreover, the specific algal carotenoid fucoxanthin, combined with pomegranate seed oil, has been demonstrated to effectively promote weight loss, decrease fat, and reduce liver fat content [42].

Macroalgae have also been investigated for their health benefits. For instance, *Laminaria japonica* (kelp), administered as a whole seaweed biomass tablet, has been shown to decrease body fat percentage in overweight Japanese men [43].

Type 2 diabetes mellitus (T2Dm) is a prevalent comorbidity of obesity, representing a significant global health concern with a rising prevalence. In 2021, the prevalence of T2Dm was estimated at 10.5%. It is estimated to increase to 11.3% by 2030 and 12.2% by 2040 [44]. Metabolic disorders, such as insulin resistance, which is frequently initiated by overweight and obesity, are considered key factors contributing to the development of T2Dm.

Brown seaweed, which is abundant in a wide variety of bioactive compounds, has shown potential in addressing these metabolic issues. These compounds have been shown to improve glucose tolerance, regulate blood lipids and promote satiety. Alginate, a polysaccharide extracted from brown seaweed, has been found to significantly increase satiety while reducing hunger and prospective food consumption when administered in a relatively high dose (15 g) [45].

Phlorotannin extracted from brown seaweeds *Ascophyllum nodosum* and *Fucus vesiculosus,* have exhibited inhibitory effects on carbohydrate-hydrolyzing enzymes α-glucosidase and α-amylase. These enzymes play a major role in carbohydrate digestion and the inhibitory effect of phlorotannin can contribute to glycemic control and the regulation of insulin levels [46,47,48].

A high dose of 500 mg *Ascophyllum nodosum* and *Fucus vesiculosus* extract administered prior to a carbohydrate load in healthy participants was found to modulate post-load insulin homeostasis. This intervention significantly reduced insulin levels and improved insulin sensitivity without affecting plasma glucose [48]. A similar trend in improving insulin homeostasis was observed in trials by De Martin et al. and Derosa et al. These trials incorporated extracts from the same species in combination with hypoglycemic agent chromium picolinate in different subgroups, including prediabetic overweight, obese patients and dysglycemic patients. Reductions in body weight and waist circumferences were observed [49,50]. These findings were further confirmed by Vodouhè et al., who conducted their study without addition of chromium in this study, no additional weight loss was observed in the algae extract, which may have been obscured by the low-calorie diet and missing dysglycemia of the participants [47].

The effect of *Fucus vesiculosus* extract on the postprandial glucose level appeared undetectable in the trial conducted by Murray et al., regardless of whether a single high (2000 mg) and low (500 mg) dose was administered [51]. In contrast, supplementation with *Ecklonia cava* extract, which is rich in phlorotannin dieckol, demonstrated a reduction in postprandial plasma glucose levels after 12 weeks of intervention [52]. The differences in intervention protocols and algae species may be the underlying cause of the varying results reported.

Dried fresh brown seaweed *Ascophyllum nodosum* and *Fucus vesiculosus* appeared to have a less potent effect compared to their extracts, resulting in only a non-significant reduction in total cholesterol and increase in HDL-C. No influence on glucose levels was found [46].

In contrast to brown seaweed, supplementation with 1500 mg/d *Chlorella* for 8 weeks did not yield detectable effects on anthropometric parameters in T2Dm patients, as reported by Hosseini et al. [53]. Similarly, no significant changes in anthropometric parameters were observed in women with dysmenorrhea followed 8 weeks of *Chlorella*-supplementation [54]. However, in patients diagnosed with non-alcoholic fatty liver disease (NAFLD), supplementation of 1200 mg/d *Chlorella* for 8 weeks resulted in reduction in body weight, along with decreased serum glucose and TNF levels [55].

Additionally, a meta-analysis of 17 studies conducted by Lak et al. examined the effect of *Spirulina* supplementation on body composition. Results demonstrated that *Spirulina* supplementation with higher doses (≥2 g/d) and a duration more than 12 weeks resulted in significant reductions in body weight (weight mean difference, WMD: −1.07 kg), BMI (WMD: −0.40) and body fat percentage (WMD: −0.84%). However, no significant effect on waist circumference was observed. Subgroup analysis revealed that older and obese individuals experienced greater benefits [56]. The findings suggest that *Spirulina* can be a promising adjunct in weight management.

**Table 4:** Overview of clinical studies investigating the effect of algae on weight loss and metabolic health. Abbreviations: BMI: body mass index; LDL-C: low-density lipoprotein cholesterol; MDA: malondialdehyde; GPx: glutathione peroxidase; SOD: superoxide dismutase; ALT: alanine aminotransferase; AST: aspartate aminotransferase; HOMA-IR: homeostatic model assessment of insulin resistance; HbA1c: hemoglobin A1c; hs-CRP: high-sensitivity C-reactive protein; PGE2: prostaglandin E2; PGF2α: prostaglandin F2; T2DM: type 2 diabetes mellitus; F.v.: Fucus vesiculosis; A.n.: Ascophyllum nodosum; NR: not reported.

Algae	Preparation	Participants	Dose	Duration	Effect	Reference
*Gelidium elegans*	Extract tablets	Overweight/obese adults	1000 mg/d	12 weeks	Decrease in body weight, BMI, fat mass and visceral fat.	[40]
*Phyaeodactylum tricornutum*	Extract capsule	Sedentary overweight women	220 mg/d	12 weeks	Preservation of bone mass and increased bone density; improvements in cardio-metabolic and quality of life markers.	[41]
*Undaria pinnatifida*	Extract capsule	Obese, non-diabetic premenopausal women	2400 mg/d	16 weeks	Significant weight loss, body fat reduction, marked liver fat reduction, decreased blood pressure.	[42]
*Laminaria japonica*	Whole biomass tablet	Overweight adults	6000 mg/d	8 weeks	Body weight percentage in men, no change in women, decrease LDL-C in non-hyperlipidemic individuals.	[43]
*Laminaria hyperborean/Lessonia trabeculata*	Extract	Healthy adults	9900 mg/d (low)/15,000 mg/d (high)	Acute single day	Enhance short term satiety, reduce glycemic response in high dose.	[45]
*Porphyridium purpureum*	Extract capsule	Overweight/obese adults	900 mg/d	8 weeks	Reduced body fat mass, body fat percentage, BMI and visceral fat; decrease in LDL-C, leptin, increase in adiponectin	[46]
*Pyropia yezoensis*	Whole biomass drink	Healthy, young men	1500 mg/d	5 days	Increased time to exhaustion, lower post-exercise lactate and ammonia, decrease MDA, increase SOD and GPx.	[47]
*Ascophyllum nodosum/Fucus vesiculosus*	Whole biomass capsule	Healthy adults	500 mg	Acute single dose	Decrease in plasma insulin, increase in Cederholm insulin sensitivity index	[48]
*Fucus vesiculosis/Ascophyllum nodosum*	Extract capsule	Overweight/obese adults	712.5 mg/d (F.v.) + 37.5 mg/d (A.n.)	6 months	Decrease in fasting glucose, fasting insulin, improvement in HOMA-IR, decrease in waist circumference.	[49]
*Fucus vesiculosis/Ascophyllum nodosum*	Extract tablet	Dysglycemia Caucasian	NR	6 months	Reduction in HbA1c, fasting plasma glucose, postprandial plasma glucose and HOMA-IR, decrease in hs-CRP, TNF-α.	[50]
*Fucus vesiculosis*	Extract capsule	Healthy adults	500 mg (low)/2000 mg (high)	Acute, 30 min before meal	No significant changes.	[51]
*Ecklonia cava*	Extract tablet	Pre-diabetic adults	1500 mg/d	12 weeks	Decrease in postprandial glucose; within treatment: decrease in insulin and C-peptide	[52]
*Chlorella vulgaris*	Whole biomass capsule	T2DM patients	1500 mg/d	8 weeks	No significant changes.	[53]
*Chlorella vulgaris*	Whole biomass capsule	Young women with primary dysmenorrhea	1500 mg/d	8 weeks	Decrease in PGE2, PGF2α, hs-CRP, MDA, pain severity, pain duration and systemic symptoms.	[54]
*Chlorella vulgaris*	Whole biomass tablet	Obese adults	1200 mg/d	8 weeks	Decrease in ALT, AST, improved fasting serum glucose, insulin, HOMA, decrease in hs-CRP.	[55]

## 6. Immune Functions

The proper functioning of the immune system is crucial not only for combating bacterial and viral infections but also for maintaining overall health. Inflammation plays a central role in the pathogenesis of various diseases, including cardiovascular disorders, neurodegenerative conditions, and cancer. Algae serve as a rich source of bioactive molecules, including polyphenols, carotenoids, polysaccharides, and fatty acids, which exhibit profound anti-inflammatory and antioxidant properties, supporting their potential use in preventive and therapeutic strategies (Table 5).

Several studies have highlighted the immune-enhancing effect of *Chlorella*. For instance, the study by Nakano et al. demonstrated that administration of *Chlorella* supplements during pregnancy increased IgA levels while reducing dioxin levels in breast milk. This is particularly beneficial for nursing infants, as IgA plays an important role in targeting antigens within their digestive tract, among other functions. It has been hypothesized that bioactive compounds in *Chlorella*, such as peptides and fibers, may stimulate IgA-producing B cells in gut-associated lymphoid tissue (GALT), subsequently increasing IgA levels in breast milk via the enteromammary pathway [57].

Specific polysaccharides and glycoproteins found in algae are hypothesized to have immunostimulatory effects by promoting B cell stimulation and proliferation. This mechanism may explain the observed increase in salivary IgA levels following *Chlorella* supplementation which could potentially enhance mucosal immune function in humans [58,59,60]. Furthermore, *Chlorella* supplementation has been demonstrated to increase serum antibody titers in healthy adults aged 50 to 55 who underwent influenza vaccination [24]. Similarly, Mekabu fucoidan, a sulfated polysaccharide extracted from seaweed, demonstrated comparable effects in elderly Japanese participants, suggesting the broader potential of algae in supporting immune function [61].

*Chlorella* supplementation has been shown to modulate cytokine levels. A study in Korea demonstrated that serum concentrations of IFN-γ and IL-1β were elevated following an 8-week *Chlorella* supplementation in healthy participants, favoring a helper T lymphocyte 1 (Th1) response. Notably, IL-1β is recognized as a Th1-induced cytokine. Furthermore, elevated natural killer (NK) cell activity was observed, which positively correlated with the elevated cytokine levels [62]. In patients with chronic hepatitis C virus (HCV) infection, *Chlorella* supplementation has been demonstrated to reduce alanine aminotransferase (ALT) levels, a liver inflammation marker, levels, further highlighting its beneficial immunostimulatory effects [63]. Additionally, another species of macroalgae *Porphyra tenera* was investigated by Jung et al. who reported an increased NK-cell activity following supplementation [64].

Herpes patients have also been shown to benefit from algae-derived treatments. A proprietary preparation of Tasmanian *Undaria pinnatifida* demonstrated an inhibitory effect on the reactivation of herpes virus, as patients with latent infections remained asymptomatic during the study. Additionally, the supplementation was associated with an increased rate of healing following herpes simplex virus type 1 (HSV-1) and type 2 (HSV-2) outbreaks [65]. Similarly, iota-carrageenan extracted from red seaweed exhibited antiviral effects by reducing viral load in children with acute symptoms of common cold when administered via nasal spray [66]. In addition, fucoidan extracted from brown seaweed resulted in a 42.4% decrease in the human T-lymphotropic virus type-1 (HTLV-1) proviral load without affecting the host immune cells [67]. These findings collectively suggest that algae-derived supplements may play a beneficial role in modulating immune function and combating viral infections.

The efficacy of algae in the management of inflammatory skin conditions has also been investigated. Roach et al. reported that oral supplementation with an algal sulfated polysaccharide (sulfated xylorhamnoglucuronan SXRG84) resulted in lower pro-inflammatory cytokine levels post-intervention than post-placebo. Furthermore, 23% of the participants reported improvements in their skin conditions, as measured by the visual analogue scale (VAS) and the dermatology quality of life index (DQLI) [68]. Similarly, the topical application of a cream containing *Gracilaria* algae improved psoriasis conditions, potentially due to its inhibitory effect on pro-inflammatory cytokines [69]. Furthermore, topical applications of *Dunaliella salina* extract, which is rich in carotenoids, significantly reduced skin’s glycation scores which measures the level advanced glycation end-(AGE) products that accumulate in the skin, and sensitivity to histamine under intense solar exposure, both of which are strong contributors to skin aging [70].

The anti-inflammatory effect of microalgae oil from *Schizochytrium* sp. on rheumatoid arthritis (RA) was demonstrated by the trial conducted by Dawczynski et al. The study demonstrated that the consumption of microalgae oil significantly reduced the number of tender joints and improved the modulation of pro-inflammatory mediators. The supplementation altered the erythrocyte lipid composition by increasing DHA levels and reducing arachidonic acid (AA)/DHA and AA/EPA ratios, suggesting a shift towards an anti-inflammatory profile. This is further supported by reduced levels of proinflammatory AA-derived eicosanoids [71]. These findings provide evidence supporting the benefits of microalgae in RA, from both clinical and biochemical perspectives.

## 7. Antioxidative Activities

Oxidative stress is believed to contribute to a range of health conditions. Fatigue, for instance, can be triggered by an imbalance of reactive oxygen and nitrogen species (RONS). Excessive RONS that are not neutralized by antioxidants can lead to tissue damage, accelerate aging, and even increase cancer risk. Several algal compounds, such as carotenoids and polyphenols, are being investigated for their potential in preventing oxidative stress-related diseases (Table 6).

Okada et al. investigated the effect of dietary *Chlorella* on oxidative stress and fatigue. Serum antioxidant capacity (AC) and malondialdehyde (MDA) levels were measured as indices of oxidative stress. Under resting condition, an increase in serum AC indicated higher antioxidant levels, which could be attributed to high levels of carotenoids, such as lutein, from *Chlorella*. A reduction in MDA levels indicated a reduction in lipid peroxidation. Furthermore, supplementation with *Chlorella* did not increase the fatigue visual analog scale (F-VAS) following fatigue exercise load, indicating the potential of *Chlorella* to enhance tolerance for fatigue [72]. *Spirulina* has also been evaluated for its ability to manage mental and physical fatigue. Johnson et al. demonstrated that supplementation with *Spirulina* improved exercise output and mental performance in active, healthy participants [73]. Other studies further demonstrated that *Spirulina* could delay exhaustion in short-term, high-intensity exercise and prolonged, strenuous exercise in healthy participants [74,75]. However, this effect was not observed in patients with idiopathic fatigue, which could be caused by metabolic abnormalities [76].

In women with dysmenorrhea, *Chlorella* supplementation has been shown to decrease MDA levels, inhibit inflammatory mediators, and thereby help to control the severity and duration of dysmenorrhea pain [54].

In an elderly population, the accumulation of phospholipid hydroperoxide (PLOOH) in erythrocyte membranes is a predominant feature of senile dementia. It is hypothesized that the inhibition of lipid peroxidation by antioxidants from *Chlorella* could reduce PLOOH accumulation, thereby potentially slowing the development of dementia. A study by Miyazawa et al. demonstrated that *Chlorella* supplementation led to increased concentrations of the plasma antioxidant lutein which consequently lowered PLOOH levels [77].

Furthermore, brown algae *Ascophyllum nodosum* extract was shown to reduce DNA damage in obese participants [78]. The presence of DNA damage has been associated with insulin resistance through cellular stress response and inflammation. One of the leading causes of DNA damage is oxidative stress. In addition, DNA damage has been demonstrated to contribute to dyslipidemia and chronic inflammation. Therefore, reducing DNA damage can lead to multiple health benefits such as improvement of metabolic health, the reduction in chronic inflammation and the reduction in cancer risk.

## 8. Cognitive Functions and Depression

Neuro-degeneration has a profound impact on the quality of life, with cognitive impairments, such as memory loss, representing a typical symptom of neuro-degenerative diseases such as Alzheimer’s disease (AD). The causes of neuro-degeneration are complex, ranging from oxidative damage to the aggregation of misfolded proteins. The antioxidative and anti-inflammatory properties of various algae species have been suggested to exert neuro-protective effects against these conditions (Table 7).

*Spirulina*, known for its antioxidative properties as previously discussed, is hypothesized to have a preventative effect against AD. A 70% ethanol extract of *Spirulina maxima* has been demonstrated to improve cognitive functions in individuals with mild cognitive impairments in Korea. Significant improvements were observed in the treatment group in visual learning and visual working memory tests [79]. A comparable trend was observed in older individuals supplemented with *Phaeodactylum tricornutum* [80]. Fermented macroalgae *Laminaria japonica* has been shown to improve scores in neurophysiological tests on short-term working memory and increase antioxidant activities in older individuals [81]. Furthermore, acute postprandial cognitive functions were significantly improved by brown seaweed supplementation in healthy adults [82].

In individuals engaged in competitive gaming, supplementation with microalgae *Phaeodactylum tricornutum* extract combined with guarana, a natural source of caffeine, showed improvements in cognitive flexibility, reaction times and other functions after 30 days of use [83]. Collectively, these findings highlight the potential of algae in improving cognitive functions and performance, as well as in combating cognitive decline associated with degenerative diseases.

Depression, another psychological disorder associated with oxidative stress, has been shown to improve with algae supplementation. Patients diagnosed with a major depression disorder (MDD) who were administered *Chlorella vulgaris* experienced alleviation from both somatic and cognitive symptoms of depression and anxiety. This improvement was reflected by significant reductions in Beck Depression Inventory II (BDI-II) and Hospital Anxiety and Depression Scale (HADS) scores [84]. Additionally, in individuals diagnosed with anhedonia, *Ulva lactuca* extract improved symptoms of depression, including sleep disorders and psychomotor symptoms [85].

## 9. Sustainability Potential of Microalgae in Human Nutrition

Given their excellent nutritional properties and various health benefits, microalgae are a highly suitable candidate for integration into the human diet. However, it is their apparently lower impact on the environment than traditional foods that especially draws attention [86]. This is particularly noteworthy since the food system in its current form is a major contributor to climate change while still failing to provide sufficient and nutritious food to the global population. The combination of nutritious and sustainable properties in microalgae suggests its potential as a food source that could contribute to addressing both of these significant global challenges [87].

Microalgae are photosynthetic organisms that convert light, water, and CO_2_ into biomass, thereby removing greenhouse gases from the atmosphere and lowering the carbon footprint [88,89,90]. Their rapid growth rate and efficient resource utilization are additional advantages that make them more attractive relative to traditional crops. When cultured in optimal conditions, algae grow up to 30-times faster than conventional food crops, doubling their volume overnight and enabling frequent harvest, thereby achieving remarkable yields [89,91].

As a further advantage, microalgae do not compete with traditional food or feed crops for scarce agricultural resources, since they do not rely on fertile soils and can be cultivated on non-arable land. Moreover, microalgae can grow in brackish or seawater, limiting the reliance on freshwater resources and its depletion significantly [86,89].

Nutrient utilization is a key factor in evaluating the sustainability of food products, as the production of fertilizer is a highly energy-intensive, costly, and harmful process to the environment [88]. Microalgae require large amounts of nutrients for their growth, with nitrogen often being the limiting factor [92]. Rather than relying solely on synthetic fertilizers, microalgae can be cultivated in nutrient-rich wastewater, thereby addressing both input demands and environmental pollution simultaneously [88]. In fact, microalgae can recycle the nutrients of agricultural or industrial wastewater, using them for their growth while simultaneously removing contaminants and cleaning the water. Taken together, this suggests their high ecological value in terms of bio-fixation and bioremediation [90].

Using wastewater for the cultivation of microalgae, however, needs rigorous safety measures in advance. Especially when produced for nutritional purposes, heavy metals and pathogens are a cause of concern and need to be removed by pre-treatment of the water. Pre-treatment of wastewater, however, is not only essential in the context of consumer safety but also to allow the growth of microalgae: since light transmittance is key for photosynthetic growth, decreasing water turbidity in advance is crucial [88].

The energy intensity of microalgae production has so far been analyzed mainly by studies investigating the economic feasibility of producing microalgae as biofuel. Much of the insights generated from those studies are likely very applicable to the production of algae as a food source. The large-scale production of algal biomass requires high levels of energy, making algae production not yet economically competitive with traditional crops. Currently, two major systems are used to cultivate microalgae: raceway ponds and bioreactors. Although the energy needed to cultivate microalgae is broadly comparable to that of conventional crops, the subsequent harvesting and downstream processing stages are significantly more energy-intensive, regardless of the cultivation method used [86,93,94]. Bioreactors in particular, achieve higher productivity and enhanced biomass purity, which is particularly relevant when microalgae are used as nutraceuticals, but at the cost of far greater energy consumption, rendering production costlier and less sustainable [86,95,96,97].

Future technological advances, particularly regarding cultivation, harvesting, and processing, will be necessary to achieve cost-effective microalgae production that has the potential for broad-range adaptation [98]. Efforts are made to address this issue through advanced breeding techniques and genetic engineering tools to enhance the quality and quantity of the final product while simultaneously minimizing production costs. However, since widespread demand is paramount for achieving scale and reducing production costs, consumer acceptance is crucial for making algae-based foods more competitive [86].

To support meaningful comparisons, future research must evaluate the actual production costs of microalgal biomass compared to traditional crops within a specific food context, rather than those for biofuel applications [86,95].

Traditional agricultural systems cause enormous environmental costs, ranging from greenhouse gas emissions and topsoil erosion to the depletion of freshwater resources and nutrient pollution. While these externalities are rarely accounted for in standard economic assessments, without a significant shift towards greater sustainability, future generations will have to pay the price eventually [86]. Hopefully, microalgae will prove to be a suitable candidate for such sustainable food sources.

## 10. Consumer Acceptance

Although algae are traditionally incorporated in many East Asian cultures, they are typically consumed in low quantities and serve as minor ingredients for flavor and texture rather than major food components. In Western societies, sensory attributes such as taste and odor remain major barriers to the broader acceptance of algae and algae-derived products in food. Consumer acceptance of these products remains mixed and highly context-dependent. There is a lack of comprehensive studies examining consumer behavior and the factors that influence the acceptance of algae protein.

Sensory characteristics are regarded as a major aspect when evaluating consumer acceptance in food studies. In the case of microalgae, the greenish color and fishy, marine-like taste are main obstacles to higher acceptance, especially in Western countries. A few studies have evaluated the acceptance of algae in food products. A study by Batista et al. performed sensory analysis of microalgae *Chlorella vulgaris* and *Arthrospira platensis* containing cookies. The results indicated that microalgae are generally acceptable if incorporated into foods, but only in low amounts. The color of *C. vulgaris* cookies was preferable while in terms of smell, the *A. platensis* cookies were more favored [99]. Microalgae incorporated into other food matrices, such as pasta, have also shown general acceptance [100,101].

Furthermore, questionnaires by Lafagra et al. demonstrated that health-conscious and environmentally aware individuals showed the highest levels of acceptance, particularly when the products are marketed with claims on the basis of sustainability [102].

Algae are generally perceived as healthy novel food in Western countries. Nevertheless, more information and clear labeling could further enhance consumer acceptance. In addition, processing steps that could reduce the unpleasant color and smell would be key steps to increase consumer acceptance. Different approaches have been proposed regarding the processing of algae products, including selective extraction and fractionation to isolate targeted bioactive components [103], enzymatic treatment [104] and controlled fermentation [105] to modify compounds responsible for off-flavors. For the majority of studies cited in this review, algae were administered in capsules, presumably to mask unpleasant color or odor for study participants. Furthermore, downstream processing strategies, such as membrane filtration and lipid removal may enable the production of algae products with improved sensory profiles. In particular, protein extraction and fractionation processes have been shown to yield protein rich products while improving flavor and reducing pigmentation [106,107].

## 11. Conclusions

The diverse health benefits of algae supplementation have been supported by numerous clinical trials which highlight their significant potential as functional foods and natural therapeutic agents. Algae, encompassing microalgae such as *Spirulina maxima*, *Chlorella vulgaris*, and *Phaeodactylum tricornutum*, as well as macroalgae like *Laminaria japonica* and *Ulva lactuca*, serve as rich sources of nutrients and bioactive compounds. These include essential nutrients, antioxidants, polyphenols, and polysaccharides. Beyond their nutritional value, these compounds provide a wide range of positive health effects. These marine organisms have been utilized to promote cardiovascular health, assist with weight management, enhance cognitive functions and support the immune system.

Although the findings from clinical trials consistently support the potential of algae as a multifunctional supplement for addressing a wide range of health challenges, several gaps remain to be addressed. The high degree of heterogeneity among studies—based on variations in study designs, statistical methods, baseline characteristics of participants and other factors—complicates the interpretation of the results. Additionally, differences in algae species and extraction methods make it challenging to standardize recommendations or fully understand the underlying mechanisms of action. Moreover, most clinical trials have focused on short-term outcomes, leaving long-term safety and efficacy largely unexplored.

Future research should prioritize conducting larger, long-term, studies to validate these existing findings and optimize algae-based interventions. Establishing more standardized frameworks for such studies is also essential to ensure more consistent and comparable outcomes. In addition, development and optimization of processing, extraction and formulation strategies that improve sensory profiles of algae products while maintaining nutritional quality is key to broadening the acceptance of algae and algae-derived products at nutritionally relevant levels in everyday foods.

In conclusion, algae represent a versatile and sustainable resource with immense potential to improve human health. Their wide-ranging benefits, from cardiovascular and metabolic health to immune and cognitive function, highlight their value as both a functional food and a therapeutic agent. As research continues to expand, algae could play an important role in promoting health and addressing chronic diseases, offering a natural and holistic approach to improving quality of life.

## Figures and Tables

**Table 1:** Comparative nutritional composition of algae vs. other protein sources (average % dry matter [3]).

Source	Protein (%)	Carbohydrates (%)	Lipids (%)
*Chlorella vulgaris*	38	33	5
*Spirulina platensis*	52	15	3
Soybean	37	30	20
egg	47	4	41

**Table 2:** Overview of studies investigating the effect of algae on lipoprotein metabolism. Abbreviations: HDL-C: high-density-lipoprotein cholesterol; LDL-C: low-density-lipoprotein cholesterol VLDL-C: very-low-density-lipoprotein cholesterol; EPA: eicosapentaenoic acid; DHA: docosahexaenoic acid; EBC: red blood cell; PE: phosphatidylethanolamine; PC: phosphatidylcholine; PL: plasma phospholipid; NR: not reported.

Algae	Preparation	Participants	Dose	Duration	Effect	Reference
*Fucus vesiculosus*	Powdered extract	Overweight/obese adults with elevated LDL-C	2000 mg/d	12 weeks	Increase in HDL-C (+9.5%), no change in LDL-C, total glycerol, triglyceride, glucose, insulin or inflammatory markers.	[26]
*Nannochloropsis* sp.	Ethanol extract capsule	Healthy adults	1000 mg/d	12 weeks	Increase in omega-3 index (+16%), decrease in VLDL-C (−25%), decrease in total glycerol (−3%), no change in triglyceride, LDL-C, small reductions in body weight and hip circumference.	[27]
*Schizochytrium* sp.	Oil extract capsule	Mild to moderate hypertriglyceridemia	4000 mg/d	14 weeks	Significant decrease in TAG (−18.9%), increase LDL-C (+4.6%), HDL-C (+4.3%), no significant change in total-C, increase in plasma EPA, DHA.	[28]
*Schizochytrium* sp.	Extract oil capsule	Healthy adults	625 mg/d	3 h following ingestion	Increase in serum DHA level with largest increase in vegan (+124%), lacto-ovo-vegetarian (+59%) and omnivore (+24%).	[29]
*Ulkenia* sp.	Extract oil capsule	Healthy vegetarian	2280 mg/d	8 weeks	Increase in RBC DHA, PE, PC, increase in PL, increase in omega-3 index.	[30]
*Phaeodactylum tricornutum*	Whole biomass in water	Healthy adults	5300 mg/d	2 weeks	Increase in total LCn-3 PUFA, increase in plasma EPA, no change in DHA.	[31]
*Chlorella pyrenoidosa, Microchloropsis salina*	Whole biomass smoothie	Healthy adults	15,000 mg/d	14 days	Chlorella decreased total cholesterol, LDL-C, HDL-C, Microchlorosis increased plasma EPA and EPA.	[32]
*Ulkenia* sp.	Extract oil capsule	Healthy vegetarians	2280 mg/d	8 weeks	Decrease in plasma triglyceride (−23%), increase in total cholesterol, LDL-C, HDL-C.	[33]
*Chlorella vulgaris*	Whole biomass tablet	Mildly hypercholesterolemic adults	5000 mg/d	4 weeks	Decrease in serum total cholesterol (−1.6%), triglyceride (−10.3%), VLDL-C (−11%).	[34]
*Chlorella vulgaris*	Whole biomass tablet	Healthy adults	5000 mg/d	4 weeks	Chlorella prevented serum total cholesterol and LDL-C rise after cholesterol challenge.	[35]

**Table 3:** Overview of the studies investigating the effect of algae on hypertension. Abbreviations: SBP: systolic blood pressure; DBP: diastolic blood pressure.

Algae	Preparation	Participants	Dose	Duration	Effect	Reference
*Pyropia yezoensis*	Whole biomass roasted sheets	Healthy preschool children	1760 mg/d	10 weeks	Significant decrease in DBP in boys and no difference of SBP and DBP in girls.	[37]
*Undaria pinnatifida*	Whole biomass in capsule	Elderly with hypertension	3300 mg/d	8 weeks	Decrease in SBP and DBP, decrease in total cholesterol in hypercholestolemic subgroups (−8%).	[38]

**Table 5:** Overview of the studies investigating the effect of algae on immune function. Abbreviations: sIgA: salivary immunoglobulin A; NK cell: natural killer cell; ALT: alanine aminotransferase; AST: aspartate aminotransferase; HAM/TSP: HTLV-1-associated myelopathy/tropical spastic paraparesis; PASI: psoriasis area and severity index; PGA: physician global assessment.

Algae	Preparation	Participants	Dose	Duration	Effect	Reference
*Chlorella pyrenoidosa*	Whole biomass tablet	Pregnant women	NR	Throughout pregnancy	Lower breast-milk dioxin and higher Ig-A levels in Chlorella group	[57]
*Chlorella pyrenoidosa*	Whole biomass tablet	Healthy, physically active	6000 mg/d	4 weeks	Increase in resting, non-exercise state sIgA	[58]
*Chlorella pyrenoidosa*	Whole biomass tablet	Female keno athletes	6000 mg/d	4 weeks	Chlorella attenuated sIgA decline during intense kendo training	[59]
*Chlorella pyrenoidosa*	Whole biomass tablet	Healthy men	6000 mg	4 weeks	Increase sIgA concentration and secretion rate	[60]
*Chlorella pyrenoidosa*	Extract in capsule	Healthy adults	600 mg/d	28 days	No enhancement in influenza antibody response overall, but improved response in participants ≤ 55 years	[24]
*Undaria pinnatifida*	Extract	Elderly adults	1800 mg/d	24 weeks	Enhanced antibody titers and preserved NK cell activity	[61]
*Chlorella vulgaris*	Whole biomass tablet	Healthy adults	5000 mg/d	8 weeks	Enhanced NK cell activity, increase in IFN-γ, IL-1β, IL-12	[62]
*Chlorella pyrenoidosa*	Whole biomass tablet and extract	Chronic HCV genotype 1 patients	3000 mg/d (1–7 d); 4500 mg/d (2–12 w); Extract: 6000 mg/d	12 weeks	Decrease in ALT level (11/13 patients), decrease AST level (9/13 patients), improved subjective well-being	[63]
*Porphyra tenera*	Extract	Healthy adults	2500 mg/d	8 weeks	Increased NK cell activity	[64]
*Undaria pinnatifida*	Whole biomass	Herpes patients	Active infection: 22,400 mg/d; Maintenance 1120 mg/d	Active phase: 10 days, maintenance up to 24 months	Active infection patients experienced faster lesion healing and reduced pain; latent patients had complete inhibition of outbreaks during maintenance period	[65]
*Red seaweed (species NR)*	Nasal spray	Children with acute phase symptoms of cold for <48 h	0.84 mL/d	7 days	Decrease in time to symptom clearance, decrease in viral load, prevention of secondary viral infections	[66]
*Cladosiphon okamuranus*	Extract	HAM/TSP patients	6000 mg/d	6–13 months	Decrease in HTLV-1 proviral load (42.4%)	[67]
*Gracilaria* sp.	Extract	Adults with inflammatory skin conditions	2000 mg	6 weeks	Improved skin symptoms	[68]
*Gracilaria* sp.	Whole biomass cream	Mild to moderate plaque psoriasis patients	Once daily	8 weeks	Improved PASI and PGA	[69]
*Dunaliella salina*	Extract cream	Female with intense sun exposure	1% extract in cream	56 days	Reduction in skin glycation, inflammation, wrinkles and redness, improve in skin reactivity	[70]
*Laminaria japonica*	Extract	Mild to moderate atopic dermatitis patients	1000 mg/d	8 weeks	Decrease in scoring atopic dermatitis, transepidermal water loss and increase skin hydration, improved clinical symptoms	[71]

**Table 6:** Overview of studies investigating the antioxidant activities of algae. Abbreviations: MDA: malondialdehyde; GHS: glutathione; TBARS: thiobarbituric acid-reactive substance; LDH: lactate dehydrogenase; SOD: superoxide dismutase; GPx: glutathione peroxidase; CK: creatine kinase; TOC: total oxidative capacity; PLOOH: phospholipid hydroperoxide.

Algae	Preparation	Participants	Dose	Duration	Effect	Reference
*Parachlorella beijerinckii*	Whole biomass tablet	Healthy male	6000 mg/d	4 weeks	Increased antioxidant capacity and decrease in MDA in resting condition	[72]
*Spirulina platensis*	Whole biomass tablet	Healthy male	3000 mg/d	8 weeks	Increase in exercise output on cross trainer machine after 1-week supplementation; improvement in Uchida–Kraepelin test (UKT) after 4-week and 8-week supplementation; improvement in Multidimensional Assessment of Fatigue Test	[73]
*Spirulina platensis*	Whole biomass capsule	Moderately trained male	6000 mg/d	4 weeks	Increased time to fatigue, decreased carbohydrate oxidation rate, increased fat oxidation rate; higher GSH levels; no increase in TBARS level after exercise	[74]
*Spirulina platensis*	Whole biomass capsule	Healthy adults	7500 mg/d	3 weeks	Decreased MDA level, LDH; larger increases in OSD, GPx; decrease in CK (−28.8%); increased time to exhaustion	[75]
*Spirulina platensis*	Whole biomass capsule	Idiopathic chronic fatigue patient	3000 mg/d	4 weeks	No difference in scores of fatigue (self-evaluated)	[76]
*Chlorella pyrenoidosa*	Whole biomass tablet	Healthy senior	8000 mg/d	2 months	Decrease in erythrocyte PLOOH, increase in erythrocyte and plasma lutein	[77]
*Ascophyllum nodosum*	Extract	Overweight/obese adults	400 mg/d	8 weeks	Decrease in basal DNA damage in obese group, decrease in TOC in women	[78]

**Table 7:** Overview of studies investigating the effect of algae on cognitive function and depression. Abbreviations: BDI-II: Beck depression inventory II; HADS: hospital anxiety and depression scale.

Algae	Preparation	Participants	Dose	Duration	Effect	Reference
*Spirulina maxima*	Extract	Mild cognitive impaired patients	1000 mg/d	12 weeks	Enhanced visual learning and visual memory test results	[79]
*Phaeodactylum tricornutum*	Extract	Cognitive and memory declined patients	1100 mg/d (8.8 mg fucoxanthin)	12 weeks	Improved word recall, picture recognition reaction time, Stroop color-word test, choice reaction time, and digit vigilance test variables	[80]
*Laminaria japonica A.*	Fermented	Senior participants	1500 mg/d	6 weeks	improved neuropsychological test scores, including higher scores in the K-MMSE, numerical memory test, Raven test, and iconic memory; increased antioxidant activity	[81]
*Ascophyllum nodosum and Fucus vesiculosus*	Extract	Healthy	500 mg	3 h following ingestion	improvements to accuracy on digit vigilance and choice reaction time tasks	[82]
*Phaeodactylum tricornutum*	Extract	Experienced gamer	4.40 mg + 500 mg guarana 8.80 mg + 500 mg guarana	30 days	improved reaction times, reasoning, learning, executive control, attention shifting (cognitive flexibility), and impulsiveness	[83]
*Chlorella vulgaris*	Extract	Major depressive disorder patients	1800 mg/d	6 weeks	reductions in total and subscale BDI-II and HADS scores as well as individual subscales of depression and anxiety	[84]
*Ulva lactuca*	Extract	Anhedonia patients	6.45 mg/kg Body weight per day	12 weeks	improvement in sleep disorders, psychomotor consequences and nutrition decreased behavior Quick Inventory of Depressive Symptomatology—Self Report	[85]

## Data Availability

No new data were created or analyzed in this study. Data sharing is not applicable to this article.

## References

[1] Godfray H.C.J., Beddington J.R., Crute I.R., Haddad L., Lawrence D., Muir J.F., Pretty J., Robinson S., Thomas S.M., Toulmin C. (2010). Food Security: The Challenge of Feeding 9 Billion People. Science.

[2] Bleakley S., Hayes M. (2017). Algal Proteins: Extraction, Application, and Challenges Concerning Production. Foods.

[3] Sousa I., Gouveia L., Batista A., Raymundo A., Konstantinos N., Papadopoulus P.P. (2008). Microalgae in Novel Food Products. Food Chemistry Research Developments.

[4] Puertas G., Vázquez M. (2021). Evaluation of the Composition and Functional Properties of Whole Egg Plasma Obtained by Centrifugation. Int. J. Food Sci. Technol..

[5] Holdt S.L., Kraan S. (2011). Bioactive Compounds in Seaweed: Functional Food Applications and Legislation. J. Appl. Phycol..

[6] Angell A.R., Mata L., De Nys R., Paul N.A. (2016). The Protein Content of Seaweeds: A Universal Nitrogen-to-Protein Conversion Factor of Five. J. Appl. Phycol..

[7] García Á., Toro-Román V., Siquier-Coll J., Bartolomé I., Muñoz D., Maynar-Mariño M. (2022). Effects of Tetraselmis Chuii Microalgae Supplementation on Anthropometric, Hormonal and Hematological Parameters in Healthy Young Men: A Double-Blind Study. Int. J. Environ. Res. Public. Health.

[8] Stiefvatter L., Frick K., Lehnert K., Vetter W., Montoya-Arroyo A., Frank J., Schmid-Staiger U., Bischoff S.C. (2022). Potentially Beneficial Effects on Healthy Aging by Supplementation of the EPA-Rich Microalgae *Phaeodactylum tricornutum* or Its Supernatant—A Randomized Controlled Pilot Trial in Elderly Individuals. Mar. Drugs.

[9] Roach L.A., Meyer B.J., Fitton J.H., Winberg P. (2022). Improved Plasma Lipids, Anti-Inflammatory Activity, and Microbiome Shifts in Overweight Participants: Two Clinical Studies on Oral Supplementation with Algal Sulfated Polysaccharide. Mar. Drugs.

[10] Teas J., Braverman L.E., Kurzer M.S., Pino S., Hurley T.G., Hebert J.R. (2007). Seaweed and Soy: Companion Foods in Asian Cuisine and Their Effects on Thyroid Function in American Women. J. Med. Food.

[11] Aquaron R., Delange F., Marchal P., Lognoné V., Ninane L. (2002). Bioavailability of Seaweed Iodine in Human Beings. Cell. Mol. Biol. Noisy Gd. Fr..

[12] Noahsen P., Kleist I., Larsen H.M., Andersen S. (2020). Intake of Seaweed as Part of a Single Sushi Meal, Iodine Excretion and Thyroid Function in Euthyroid Subjects: A Randomized Dinner Study. J. Endocrinol. Investig..

[13] Clark C.D., Bassett B., Burge M.R. (2003). Effects of Kelp Supplementation on Thyroid Function in Euthyroid Subjects. Endocr. Pract..

[14] Boye J., Zare F., Pletch A. (2010). Pulse Proteins: Processing, Characterization, Functional Properties and Applications in Food and Feed. Food Res. Int..

[15] Caporgno M.P., Mathys A. (2018). Trends in Microalgae Incorporation into Innovative Food Products with Potential Health Benefits. Front. Nutr..

[16] Becker E.W. (2007). Micro-Algae as a Source of Protein. Biotechnol. Adv..

[17] Tibbetts S., Patelakis S. (2021). Apparent Digestibility Coefficients (ADCs) of Intact-Cell Marine Microalgae Meal (*Pavlova* sp. 459) for Juvenile Atlantic Salmon (*Salmo salar* L.). Aquaculture.

[18] Suo Q., Yue Y., Wang J., Wu N., Geng L., Zhang Q. (2022). Isolation, Identification and in Vivo Antihypertensive Effect of Novel Angiotensin I-Converting Enzyme (ACE) Inhibitory Peptides from Spirulina Protein Hydrolysate. Food Funct..

[19] Shahraki Jazinaki M., Rashidmayvan M., Rahbarinejad P., Shadmand Foumani Moghadam M.R., Pahlavani N. (2025). Effects of Spirulina Supplementation on C-Reactive Protein (CRP): A Systematic Review and Dose-Response Meta-Analysis. Food Sci. Nutr..

[20] van der Heijden I., West S., Monteyne A.J., Finnigan T.J.A., Abdelrahman D.R., Murton A.J., Stephens F.B., Wall B.T. (2023). Algae Ingestion Increases Resting and Exercised Myofibrillar Protein Synthesis Rates to a Similar Extent as Mycoprotein in Young Adults. J. Nutr..

[21] La Mantia I., Maniaci A., Scibilia G., Scollo P. (2024). Effects of a Dietary Microalgae (*Arthrospira platensis*) Supplement on Stress, Well-Being, and Performance in Water Polo Players: A Clinical Case Series. Nutrients.

[22] Toro V., Siquier-Coll J., Bartolomé I., Robles-Gil M.C., Rodrigo J., Maynar-Mariño M. (2020). Effects of Tetraselmis Chuii Microalgae Supplementation on Ergospirometric, Haematological and Biochemical Parameters in Amateur Soccer Players. Int. J. Environ. Res. Public. Health.

[23] Merchant R.E., Carmack C.A., Wise C.M. (2000). Nutritional Supplementation with *Chlorella pyrenoidosa* for Patients with Fibromyalgia Syndrome: A Pilot Study. Phytother. Res..

[24] Halperin S.A., Smith B., Nolan C., Shay J., Kralovec J. (2003). Safety and Immunoenhancing Effect of a Chlorella-Derived Dietary Supplement in Healthy Adults Undergoing Influenza Vaccination: Randomized, Double-Blind, Placebo-Controlled Trial. CMAJ Can. Med. Assoc. J..

[25] Shimada M., Hasegawa T., Nishimura C., Kan H., Kanno T., Nakamura T., Matsubayashi T. (2009). Anti-Hypertensive Effect of γ-Aminobutyric Acid (GABA)-Rich Chlorella on High-Normal Blood Pressure and Borderline Hypertension in Placebo-Controlled Double Blind Study. Clin. Exp. Hypertens..

[26] Murray M., Dordevic A.L., Cox K., Scholey A., Ryan L., Bonham M.P. (2021). Twelve Weeks’ Treatment with a Polyphenol-Rich Seaweed Extract Increased HDL Cholesterol with No Change in Other Biomarkers of Chronic Disease Risk in Overweight Adults: A Placebo-Controlled Randomized Trial. J. Nutr. Biochem..

[27] Rao A., Briskey D., Nalley J.O., Ganuza E. (2020). Omega-3 Eicosapentaenoic Acid (EPA) Rich Extract from the Microalga *Nannochloropsis* Decreases Cholesterol in Healthy Individuals: A Double-Blind, Randomized, Placebo-Controlled, Three-Month Supplementation Study. Nutrients.

[28] Maki K.C., Yurko-Mauro K., Dicklin M.R., Schild A.L., Geohas J.G. (2014). A New, Microalgal DHA- and EPA-Containing Oil Lowers Triacylglycerols in Adults with Mild-to-Moderate Hypertriglyceridemia. Prostaglandins Leukot. Essent. Fatty Acids.

[29] García-Maldonado E., Alcorta A., Zapatera B., Vaquero M.P. (2023). Changes in Fatty Acid Levels after Consumption of a Novel Docosahexaenoic Supplement from Algae: A Crossover Randomized Controlled Trial in Omnivorous, Lacto-Ovo Vegetarians and Vegans. Eur. J. Nutr..

[30] Geppert J., Kraft V., Demmelmair H., Koletzko B. (2005). Docosahexaenoic Acid Supplementation in Vegetarians Effectively Increases Omega-3 Index: A Randomized Trial. Lipids.

[31] Stiefvatter L., Lehnert K., Frick K., Montoya-Arroyo A., Frank J., Vetter W., Schmid-Staiger U., Bischoff S.C. (2021). Oral Bioavailability of Omega-3 Fatty Acids and Carotenoids from the Microalgae *Phaeodactylum tricornutum* in Healthy Young Adults. Mar. Drugs.

[32] Sandgruber F., Höger A.-L., Kunze J., Schenz B., Griehl C., Kiehntopf M., Kipp K., Kühn J., Stangl G.I., Lorkowski S. (2023). Impact of Regular Intake of Microalgae on Nutrient Supply and Cardiovascular Risk Factors: Results from the NovAL Intervention Study. Nutrients.

[33] Geppert J., Kraft V., Demmelmair H., Koletzko B. (2006). Microalgal Docosahexaenoic Acid Decreases Plasma Triacylglycerol in Normolipidaemic Vegetarians: A Randomised Trial. Br. J. Nutr..

[34] Ryu N.H., Lim Y., Park J.E., Kim J., Kim J.Y., Kwon S.W., Kwon O. (2014). Impact of Daily Chlorella Consumption on Serum Lipid and Carotenoid Profiles in Mildly Hypercholesterolemic Adults: A Double-Blinded, Randomized, Placebo-Controlled Study. Nutr. J..

[35] Kim S., Kim J., Lim Y., Kim Y.J., Kim J.Y., Kwon O. (2016). A Dietary Cholesterol Challenge Study to Assess Chlorella Supplementation in Maintaining Healthy Lipid Levels in Adults: A Double-Blinded, Randomized, Placebo-Controlled Study. Nutr. J..

[36] Pinto-Leite M., Martins D., Ferreira A.C., Silva C., Trindade F., Saraiva F., Vitorino R., Barros R., Lima P.A., Leite-Moreira A. (2025). The Role of Chlorella and Spirulina as Adjuvants of Cardiovascular Risk Factor Control: A Systematic Review and Meta-Analysis of Randomised Controlled Trials. Nutrients.

[37] Wada K., Tsuji M., Nakamura K., Oba S., Nishizawa S., Yamamoto K., Watanabe K., Ando K., Nagata C. (2021). Effect of Dietary Nori (Dried Laver) on Blood Pressure in Young Japanese Children: An Intervention Study. J. Epidemiol..

[38] Hata Y., Nakajima K., Uchida J., Hidaka H., Nakano T. (2001). Clinical Effects of Brown Seaweed, *Undaria pinnatifida* (Wakame), on Blood Pressure in Hypertensive Subjects. J. Clin. Biochem. Nutr..

[39] Emmerich S., Fryar C., Stierman B., Ogden C. (2024). Obesity and Severe Obesity Prevalence in Adults: United States, August 2021–August 2023.

[40] Kim C.O., Kim Y.N., Lee D.-C. (2019). Effects of Gelidium Elegans on Weight and Fat Mass Reduction and Obesity Biomarkers in Overweight or Obese Adults: A Randomized Double-Blinded Study. Nutrients.

[41] Dickerson B., Maury J., Jenkins V., Nottingham K., Xing D., Gonzalez D.E., Leonard M., Kendra J., Ko J., Yoo C. (2024). Effects of Supplementation with Microalgae Extract from *Phaeodactylum tricornutum* (Mi136) to Support Benefits from a Weight Management Intervention in Overweight Women. Nutrients.

[42] Abidov M., Ramazanov Z., Seifulla R., Grachev S. (2010). The Effects of Xanthigen^TM^ in the Weight Management of Obese Premenopausal Women with Non-Alcoholic Fatty Liver Disease and Normal Liver Fat. Diabetes Obes. Metab..

[43] Aoe S., Yamanaka C., Ohtoshi H., Nakamura F., Fujiwara S. (2021). Effects of Daily Kelp (*Laminaria japonica*) Intake on Body Composition, Serum Lipid Levels, and Thyroid Hormone Levels in Healthy Japanese Adults: A Randomized, Double-Blind Study. Mar. Drugs.

[44] Ye J., Wu Y., Yang S., Zhu D., Chen F., Chen J., Ji X., Hou K. (2023). The Global, Regional and National Burden of Type 2 Diabetes Mellitus in the Past, Present and Future: A Systematic Analysis of the Global Burden of Disease Study 2019. Front. Endocrinol..

[45] Georg M.G., Kristensen M., Belza A., Knudsen J.C., Astrup A. (2012). Acute Effect of Alginate-Based Preload on Satiety Feelings, Energy Intake, and Gastric Emptying Rate in Healthy Subjects. Obesity.

[46] Geurts K.A.M., Meijer S., Roeters van Lennep J.E., Wang X., Özcan B., Voortman G., Liu H., Castro Cabezas M., Berk K.A., Mulder M.T. (2024). The Effect of Sargassum Fusiforme and *Fucus vesiculosus* on Continuous Glucose Levels in Overweight Patients with Type 2 Diabetes Mellitus: A Feasibility Randomized, Double-Blind, Placebo-Controlled Trial. Nutrients.

[47] Vodouhè M., Marois J., Guay V., Leblanc N., Weisnagel S.J., Bilodeau J.-F., Jacques H. (2022). Marginal Impact of Brown Seaweed Ascophyllum Nodosum and *Fucus vesiculosus* Extract on Metabolic and Inflammatory Response in Overweight and Obese Prediabetic Subjects. Mar. Drugs.

[48] Paradis M.-E., Couture P., Lamarche B. (2011). A Randomised Crossover Placebo-Controlled Trial Investigating the Effect of Brown Seaweed (*Ascophyllum nodosum* and *Fucus vesiculosus*) on Postchallenge Plasma Glucose and Insulin Levels in Men and Women. Appl. Physiol. Nutr. Metab..

[49] De Martin S., Gabbia D., Carrara M., Ferri N. (2018). The Brown Algae *Fucus vesiculosus* and *Ascophyllum nodosum* Reduce Metabolic Syndrome Risk Factors: A Clinical Study. Nat. Prod. Commun..

[50] Derosa G., Cicero A.F.G., D’Angelo A., Maffioli P. (2019). *Ascophyllum nodosum* and *Fucus vesiculosus* on Glycemic Status and on Endothelial Damage Markers in Dysglicemic Patients. Phytother. Res..

[51] Murray M., Dordevic A.L., Ryan L., Bonham M.P. (2018). The Impact of a Single Dose of a Polyphenol-Rich Seaweed Extract on Postprandial Glycaemic Control in Healthy Adults: A Randomised Cross-Over Trial. Nutrients.

[52] Lee S.-H., Jeon Y.-J. (2015). Efficacy and Safety of a Dieckol-Rich Extract (AG-Dieckol) of Brown Algae, Ecklonia Cava, in Pre-Diabetic Individuals: A Double-Blind, Randomized, Placebo-Controlled Clinical Trial. Food Funct..

[53] Hosseini A.M., Keshavarz S.A., Nasli-Esfahani E., Amiri F., Janani L. (2021). The Effects of Chlorella Supplementation on Glycemic Control, Lipid Profile and Anthropometric Measures on Patients with Type 2 Diabetes Mellitus. Eur. J. Nutr..

[54] Haidari F., Homayouni F., Helli B., Haghighizadeh M.H., Farahmandpour F. (2018). Effect of Chlorella Supplementation on Systematic Symptoms and Serum Levels of Prostaglandins, Inflammatory and Oxidative Markers in Women with Primary Dysmenorrhea. Eur. J. Obstet. Gynecol. Reprod. Biol..

[55] Ebrahimi-Mameghani M., Sadeghi Z., Farhangi M.A., Vaghef-Mehrabany E., Aliashrafi S. (2017). Glucose Homeostasis, Insulin Resistance and Inflammatory Biomarkers in Patients with Non-Alcoholic Fatty Liver Disease: Beneficial Effects of Supplementation with Microalgae Chlorella Vulgaris: A Double-Blind Placebo-Controlled Randomized Clinical Trial. Clin. Nutr..

[56] Lak M., Karimi M., Akhgarjand C., Ghotboddin Mohammadi S., Pam P., Ashtary-Larky D., Pirzad S., Amirkhan-Dehkordi M., Shahrbaf M.A., Henselmans M. (2025). Effects of Spirulina Supplementation on Body Composition in Adults: A GRADE-Assessed and Dose–Response Meta-Analysis of RCTs. Nutr. Metab..

[57] Nakano S., Takekoshi H., Nakano M. (2007). Chlorella (*Chlorella pyrenoidosa*) Supplementation Decreases Dioxin and Increases Immunoglobulin A Concentrations in Breast Milk. J. Med. Food.

[58] Chidley C., Davison G. (2018). The Effect of *Chlorella pyrenoidosa* Supplementation on Immune Responses to 2 Days of Intensified Training. Eur. J. Nutr..

[59] Otsuki T., Shimizu K., Iemitsu M., Kono I. (2012). Chlorella Intake Attenuates Reduced Salivary SIgA Secretion in Kendotraining Camp Participants. Nutr. J..

[60] Otsuki T., Shimizu K., Iemitsu M., Kono I. (2011). Salivary Secretory Immunoglobulin a Secretion Increases after 4-Weeks Ingestion of Chlorella-Derived Multicomponent Supplement in Humans: A Randomized Cross over Study. Nutr. J..

[61] Negishi H., Mori M., Mori H., Yamori Y. (2013). Supplementation of Elderly Japanese Men and Women with Fucoidan from Seaweed Increases Immune Responses to Seasonal Influenza Vaccination. J. Nutr..

[62] Kwak J.H., Baek S.H., Woo Y., Han J.K., Kim B.G., Kim O.Y., Lee J.H. (2012). Beneficial Immunostimulatory Effect of Short-Term Chlorella Supplementation: Enhancement of Natural Killercell Activity and Early Inflammatory Response (Randomized, Double-Blinded, Placebo-Controlled Trial). Nutr. J..

[63] Azocar J., Diaz A. (2013). Efficacy and Safety of Chlorella Supplementation in Adults with Chronic Hepatitis C Virus Infection. World J. Gastroenterol..

[64] Jung S.-J., Jang H.-Y., Jung E.-S., Noh S.-O., Shin S.-W., Ha K.-C., Baek H.-I., Ahn B.-J., Oh T.-H., Chae S.-W. (2020). Effects of Porphyra Tenera Supplementation on the Immune System: A Randomized, Double-Blind, and Placebo-Controlled Clinical Trial. Nutrients.

[65] Cooper R., Dragar C., Elliot K., Fitton J., Godwin J., Thompson K. (2002). GFS, a Preparation of Tasmanian *Undaria pinnatifida* Is Associated with Healing and Inhibition of Reactivation of Herpes. BMC Complement. Altern. Med..

[66] Fazekas T., Eickhoff P., Pruckner N., Vollnhofer G., Fischmeister G., Diakos C., Rauch M., Verdianz M., Zoubek A., Gadner H. (2012). Lessons Learned from a Double-Blind Randomised Placebo-Controlled Study with a Iota-Carrageenan Nasal Spray as Medical Device in Children with Acute Symptoms of Common Cold. BMC Complement. Altern. Med..

[67] Araya N., Takahashi K., Sato T., Nakamura T., Sawa C., Hasegawa D., Ando H., Aratani S., Yagishita N., Fujii R. (2011). Fucoidan Therapy Decreases the Proviral Load in Patients with Human T-Lymphotropic Virus Type-1-Associated Neurological Disease. Antivir. Ther..

[68] Roach L.A., Meyer B.J., Fitton J.H., Winberg P. (2023). Oral Supplementation with Algal Sulphated Polysaccharide in Subjects with Inflammatory Skin Conditions: A Randomised Double-Blind Placebo-Controlled Trial and Baseline Dietary Differences. Mar. Drugs.

[69] Shatalebi M.A., Bokaie Jazi S., Yegdaneh A., Iraji F., Siadat A.H., Noorshargh P. (2020). Comparative Evaluation of Gracilaria Algae 3% Cream vs Clobetasol 0.05% Cream in Treatment of Plaque Type Psoriasis: A Randomized, Split-Body, Triple-Blinded Clinical Trial. Dermatol. Ther..

[70] Havas F., Krispin S., Cohen M., Loing E., Farge M., Suere T., Attia-Vigneau J. (2022). A Dunaliella Salina Extract Counteracts Skin Aging under Intense Solar Irradiation Thanks to Its Antiglycation and Anti-Inflammatory Properties. Mar. Drugs.

[71] Dawczynski C., Dittrich M., Neumann T., Goetze K., Welzel A., Oelzner P., Völker S., Schaible A.M., Troisi F., Thomas L. (2018). Docosahexaenoic Acid in the Treatment of Rheumatoid Arthritis: A Double-Blind, Placebo-Controlled, Randomized Cross-over Study with Microalgae vs. Sunflower Oil. Clin. Nutr..

[72] Okada H., Yoshida N., Kakuma T., Toyomasu K. (2017). Effect of Chlorella Ingestion on Oxidative Stress and Fatigue Symptoms in Healthy Men. Kurume Med. J..

[73] Johnson M., Hassinger L., Davis J., Devor S.T., DiSilvestro R.A. (2016). A Randomized, Double Blind, Placebo Controlled Study of Spirulina Supplementation on Indices of Mental and Physical Fatigue in Men. Int. J. Food Sci. Nutr..

[74] Kalafati M., Jamurtas A.Z., Nikolaidis M.G., Paschalis V., Theodorou A.A., Sakellariou G.K., Koutedakis Y., Kouretas D. (2010). Ergogenic and Antioxidant Effects of Spirulina Supplementation in Humans. Med. Sci. Sports Exerc..

[75] Lu H.-K., Hsieh C.-C., Hsu J.-J., Yang Y.-K., Chou H.-N. (2006). Preventive Effects of Spirulina Platensis on Skeletal Muscle Damage under Exercise-Induced Oxidative Stress. Eur. J. Appl. Physiol..

[76] Baicus C., Baicus A. (2007). Spirulina Did Not Ameliorate Idiopathic Chronic Fatigue in Four N-of-1 Randomized Controlled Trials. Phytother. Res..

[77] Miyazawa T., Nakagawa K., Takekoshi H., Higuchi O., Kato S., Kondo M., Kimura F., Miyazawa T. (2013). Ingestion of Chlorella Reduced the Oxidation of Erythrocyte Membrane Lipids in Senior Japanese Subjects. J. Oleo Sci..

[78] Baldrick F.R., McFadden K., Ibars M., Sung C., Moffatt T., Megarry K., Thomas K., Mitchell P., Wallace J.M.W., Pourshahidi L.K. (2018). Impact of a (Poly)Phenol-Rich Extract from the Brown Algae *Ascophyllum Nodosum* on DNA Damage and Antioxidant Activity in an Overweight or Obese Population: A Randomized Controlled Trial. Am. J. Clin. Nutr..

[79] Choi W.-Y., Lee W.-K., Kim T.-H., Ryu Y.-K., Park A., Lee Y.-J., Heo S.-J., Oh C., Chung Y.-C., Kang D.-H. (2022). The Effects of Spirulina Maxima Extract on Memory Improvement in Those with Mild Cognitive Impairment: A Randomized, Double-Blind, Placebo-Controlled Clinical Trial. Nutrients.

[80] Yoo C., Maury J., Gonzalez D.E., Ko J., Xing D., Jenkins V., Dickerson B., Leonard M., Estes L., Johnson S. (2024). Effects of Supplementation with a Microalgae Extract from *Phaeodactylum tricornutum* Containing Fucoxanthin on Cognition and Markers of Health in Older Individuals with Perceptions of Cognitive Decline. Nutrients.

[81] Reid S.N.S., Ryu J., Kim Y., Jeon B.H. (2018). The Effects of Fermented *Laminaria japonica* on Short-Term Working Memory and Physical Fitness in the Elderly. Evid. Based Complement. Alternat. Med..

[82] Haskell-Ramsay C.F., Jackson P.A., Dodd F.L., Forster J.S., Bérubé J., Levinton C., Kennedy D.O. (2018). Acute Post-Prandial Cognitive Effects of Brown Seaweed Extract in Humans. Nutrients.

[83] Leonard M., Maury J., Dickerson B., Gonzalez D.E., Kendra J., Jenkins V., Nottingham K., Yoo C., Xing D., Ko J. (2023). Effects of Dietary Supplementation of a Microalgae Extract Containing Fucoxanthin Combined with Guarana on Cognitive Function and Gaming Performance. Nutrients.

[84] Panahi Y., Badeli R., Karami G.-R., Badeli Z., Sahebkar A. (2015). A Randomized Controlled Trial of 6-Week *Chlorella Vulgaris* Supplementation in Patients with Major Depressive Disorder. Complement. Ther. Med..

[85] Allaert F.-A., Demais H., Collén P.N. (2018). A Randomized Controlled Double-Blind Clinical Trial Comparing versus Placebo the Effect of an Edible Algal Extract (Ulva Lactuca) on the Component of Depression in Healthy Volunteers with Anhedonia. BMC Psychiatry.

[86] Diaz C.J., Douglas K.J., Kang K., Kolarik A.L., Malinovski R., Torres-Tiji Y., Molino J.V., Badary A., Mayfield S.P. (2023). Developing Algae as a Sustainable Food Source. Front. Nutr..

[87] Willett W., Rockström J., Loken B., Springmann M., Lang T., Vermeulen S., Garnett T., Tilman D., DeClerck F., Wood A. (2019). Food in the Anthropocene: The EAT-Lancet Commission on Healthy Diets from Sustainable Food Systems. Lancet Lond. Engl..

[88] Cuellar-Bermudez S.P., Aleman-Nava G.S., Chandra R., Garcia-Perez J.S., Contreras-Angulo J.R., Markou G., Muylaert K., Rittmann B.E., Parra-Saldivar R. (2017). Nutrients Utilization and Contaminants Removal. A Review of Two Approaches of Algae and Cyanobacteria in Wastewater. Algal Res..

[89] Ullah K., Ahmad M., Sofia, Sharma V.K., Lu P., Harvey A., Zafar M., Sultana S., Anyanwu C.N. (2014). Algal Biomass as a Global Source of Transport Fuels: Overview and Development Perspectives. Prog. Nat. Sci. Mater. Int..

[90] Zhu L. (2015). Biorefinery as a Promising Approach to Promote Microalgae Industry: An Innovative Framework. Renew. Sustain. Energy Rev..

[91] Thoré E.S.J., Muylaert K., Bertram M.G., Brodin T. (2023). Microalgae. Curr. Biol..

[92] Lu Q., Zhou W., Min M., Ma X., Ma Y., Chen P., Zheng H., Doan Y.T.T., Liu H., Chen C. (2016). Mitigating Ammonia Nitrogen Deficiency in Dairy Wastewaters for Algae Cultivation. Bioresour. Technol..

[93] Najjar Y.S.H., Abu-Shamleh A. (2020). Harvesting of Microalgae by Centrifugation for Biodiesel Production: A Review. Algal Res..

[94] Ozkan A., Kinney K., Katz L., Berberoglu H. (2012). Reduction of Water and Energy Requirement of Algae Cultivation Using an Algae Biofilm Photobioreactor. Bioresour. Technol..

[95] Chew K.W., Yap J.Y., Show P.L., Suan N.H., Juan J.C., Ling T.C., Lee D.-J., Chang J.-S. (2017). Microalgae Biorefinery: High Value Products Perspectives. Bioresour. Technol..

[96] Chisti Y. (2007). Biodiesel from Microalgae. Biotechnol. Adv..

[97] Ganesh Saratale R., Ponnusamy V.K., Jeyakumar R.B., Sirohi R., Piechota G., Shobana S., Dharmaraja J., Lay C., Dattatraya Saratale G., Seung Shin H. (2022). Microalgae Cultivation Strategies Using Cost–Effective Nutrient Sources: Recent Updates and Progress towards Biofuel Production. Bioresour. Technol..

[98] Lafarga T., Acién-Fernández F.G., Castellari M., Villaró S., Bobo G., Aguiló-Aguayo I. (2019). Effect of Microalgae Incorporation on the Physicochemical, Nutritional, and Sensorial Properties of an Innovative Broccoli Soup. LWT.

[99] Batista A.P., Niccolai A., Fradinho P., Fragoso S., Bursic I., Rodolfi L., Biondi N., Tredici M.R., Sousa I., Raymundo A. (2017). Microalgae Biomass as an Alternative Ingredient in Cookies: Sensory, Physical and Chemical Properties, Antioxidant Activity and *in Vitro* Digestibility. Algal Res..

[100] Zouari N., Abid M., Fakhfakh N., Ayadi M.A., Zorgui L., Ayadi M., Attia H. (2011). Blue-Green Algae (*Arthrospira platensis*) as an Ingredient in Pasta: Free Radical Scavenging Activity, Sensory and Cooking Characteristics Evaluation. Int. J. Food Sci. Nutr..

[101] Grahl S., Strack M., Mensching A., Mörlein D. (2020). Alternative Protein Sources in Western Diets: Food Product Development and Consumer Acceptance of Spirulina-Filled Pasta. Food Qual. Prefer..

[102] Lafarga T., Rodríguez-Bermúdez R., Morillas-España A., Villaró S., García-Vaquero M., Morán L., Sánchez-Zurano A., González-López C.V., Acién-Fernández F.G. (2021). Consumer Knowledge and Attitudes towards Microalgae as Food: The Case of Spain. Algal Res..

[103] Singh S., Verma D.K., Thakur M., Tripathy S., Patel A.R., Shah N., Utama G.L., Srivastav P.P., Benavente-Valdés J.R., Chávez-González M.L. (2021). Supercritical Fluid Extraction (SCFE) as Green Extraction Technology for High-Value Metabolites of Algae, Its Potential Trends in Food and Human Health. Food Res. Int..

[104] O’ Brien R.O., Hayes M., Sheldrake G., Tiwari B., Walsh P. (2022). Macroalgal Proteins: A Review. Foods.

[105] Kadam S.U., Tiwari B.K., O’Donnell C.P. (2015). Extraction, Structure and Biofunctional Activities of Laminarin from Brown Algae. Int. J. Food Sci. Technol..

[106] Nunes E., Odenthal K., Nunes N., Fernandes T., Fernandes I.A., Pinheiro De Carvalho M.A.A. (2024). Protein Extracts from Microalgae and Cyanobacteria Biomass. Techno-Functional Properties and Bioactivity: A Review. Algal Res..

[107] Mosibo O.K., Ferrentino G., Udenigwe C.C. (2024). Microalgae Proteins as Sustainable Ingredients in Novel Foods: Recent Developments and Challenges. Foods.

